# Extreme weather events and HIV: development of a conceptual framework through qualitative interviews with people with HIV impacted by the California wildfires and their clinicians

**DOI:** 10.1186/s12889-023-15957-5

**Published:** 2023-05-25

**Authors:** Parya Saberi, Kristin Ming, Emily A. Arnold, Anna M. Leddy, Sheri D. Weiser

**Affiliations:** 1 Division of Prevention Science, Department of Medicine, University of California, San Francisco, San Francisco, CA USA; 2 Division of pulmonary and critical care medicine, University of California, San Francisco, San Francisco, CA USA; 3 Division of HIV, Infectious Diseases and Global Medicine, Department of Medicine, University of California, San Francisco, San Francisco, CA USA

**Keywords:** HIV, Extreme weather events, Climate change, Wildfire, Mental health, Qualitative research, Intersection, California

## Abstract

**Background:**

People with HIV (PWH) are disproportionately vulnerable to the impacts of wildfires, given the need for frequent access to healthcare systems, higher burden of comorbidities, higher food insecurity, mental and behavioral health challenges, and challenges of living with HIV in a rural area. In this study, we aim to better understand the pathways through which wildfires impact health outcomes among PWH.

**Methods:**

From October 2021 through February 2022, we conducted individual semi-structured qualitative interviews with PWH impacted by the Northern California wildfires and clinicians of PWH who were impacted by wildfires. The study aims were to explore the influence of wildfires on the health of PWH and to discuss measures at the individual, clinic, and system levels that helped to mitigate these impacts.

**Results:**

We interviewed 15 PWH and 7 clinicians. While some PWH felt that surviving the HIV epidemic added to their resilience against wildfires, many felt that the wildfires compounded the HIV-related traumas that they have experienced. Participants outlined five main routes by which wildfires negatively impacted their health: (1) access to healthcare (medications, clinics, clinic staff), (2) mental health (trauma; anxiety, depression, or stress; sleep disturbances; coping strategies), (3) physical health (cardiopulmonary, other co-morbidities), (4) social/economic impacts (housing, finances, community), and (5) nutrition and exercise. The recommendations for future wildfire preparedness were at the (1) individual-level (what to have during evacuation), (2) pharmacy-level (procedural, staffing), and (3) clinic- or county-level (funds and vouchers; case management; mental health services; emergency response planning; other services such as telehealth, home visits, home laboratory testing).

**Conclusions:**

Based on our data and prior research, we devised a conceptual framework that acknowledges the impact of wildfires at the community-, household-, and individual-level with implications for physical and mental health outcomes among PWH. These findings and framework can help in developing future interventions, programs, and policies to mitigate the cumulative impacts of extreme weather events on the health of PWH, particularly among individuals living in rural areas. Further studies are needed to examine health system strengthening strategies, innovative methods to improve access to healthcare, and community resilience through disaster preparedness.

**Trial registration:**

N/A.

## Background

In the past five years, the world has seen an unprecedented rise in the frequency, size, and duration of wildfires, with potentially devastating health consequences. People with HIV (PWH) have been identified as a group that is particularly vulnerable to the impacts of extreme weather events (EWEs) including wildfires [1]. In studies from African countries, EWEs, especially droughts and excess rainfall, have been associated with condomless and transactional sex, early sexual debut, violence, and migration [2–8], all risk factors for HIV acquisition and transmission. However, little is known about the health impacts of wildfires on the health of PWH [9, 10]. HIV and EWEs present two threats to human health, and research on the influence of EWEs on the health of PWH is critical.

Specifically, wildfires are a major threat to health including chronic disease morbidity and mortality such as cardiopulmonary diseases [1, 11–14]. Poor cardiopulmonary outcomes resulting from wildfires have included asthma-related hospitalizations and emergency department visits, chronic obstructive pulmonary disease, respiratory infection, cardiovascular events, lung cancer, and mortality [9, 15–21]. Wildfires have also been associated with increased mental health challenges including post-traumatic stress disorder (PTSD), anxiety, and depression [22], and with displacement and homelessness [23, 24].

While wildfires affect all residents of impacted areas, the health effects of these EWEs are more pronounced in vulnerable populations, especially older individuals, youth, those living in low-income areas, outdoor workers, and those with pre-existing cardiopulmonary conditions [25–27]. PWH are disproportionately vulnerable to the impacts of wildfires, given the need for frequent access to healthcare systems. They have a higher burden of comorbidities [28] requiring adherence to multiple daily medications [29], higher food insecurity [30], and mental and behavioral health challenges [31]. Additionally, PWH in rural areas may be particularly vulnerable given later HIV diagnosis and later antiretroviral therapy (ART) initiation when compared to those living in urban and metropolitan settings [32, 33]. Several factors may be related to these HIV-related health outcomes for PWH in rural areas including higher levels of HIV-related stigma; greater social isolation; longer distances to HIV care; limited transportation; and a lower access to clinicians with HIV expertise compared to their urban counterparts [34, 35]. These may be compounded by the low availability of reliable high-speed internet connection in home settings to conduct telehealth appointments [36], including during power outages [37].

Few studies have explored the impact of wildfires on the ability of PWH to engage in HIV-related health activities and HIV-related morbidities. Therefore, in this study, we aimed to better understand the complex pathways through which wildfires impact health outcomes among PWH. This understanding is critical to inform future research, health programs, and policies to mitigate the cumulative impacts of EWEs, such as wildfires, on the health of PWH.

## Methods

From October 2021 through February 2022, we conducted individual semi-structured qualitative interviews with PWH impacted by the California wildfires and clinicians of PWH who were impacted by wildfires. The aim of these interviews was to explore the influence of wildfires on the physical and mental health of PWH and to discuss measures at the individual, clinic, and system levels that helped to mitigate the impact of these EWEs. Wildfire impact was defined as any fire or smoke damage to home or place of receipt of healthcare, or any disruption to how one gets to and from medical care, where one receives food, or how one meets other primary care needs.

All study activities were conducted remotely. We recruited a convenience sample of PWH and clinicians of PWH by emailing education and training organizations (e.g., AIDS Education and Training Centers), directly emailing clinicians, and posting advertisement flyers at clinics in the areas impacted by the Northern California wildfires. Patient participants were PWH ≥ 18 years of age, who were impacted by the Northern California wildfires in the prior three years, and who had access to a mobile phone or computer. Clinician participants were any clinician (including physician, nurse, pharmacist, social worker, or case manager) who had provided care to PWH who were impacted by the Northern California wildfires and who had access to a mobile phone or computer. Verbal informed consent was received as approved by the University of California, San Francisco IRB (IRB #21-34596). Participants were emailed a copy of the consent.

Eligible participants completed a baseline quantitative survey using a HIPAA-compliant online survey tool. Patients were asked about their demographics, education, financial situation, living situation, number of years living with HIV, and HIV viral load (detectable or undetectable). Clinicians were asked about their demographics, profession and specialty, years providing patient care, number of patients in panel, number of patients with HIV in panel, number of patients with HIV who were impacted by the California wildfires, and whether their clinic was impacted by the California wildfires.

Two interview guides were developed for this study, one for patient interviews and one for clinician interviews. The interview guide for the patients was divided into the timeframes of prior to and during/after the wildfires and inquired about their access to healthcare (including appointments with primary care or HIV care clinician, refills, ART adherence, and laboratory testing), main barriers to healthcare, physical health, mental health, ability to obtain healthy food, financial situation, transportation, and social life. Patients were also asked about the things that helped them manage the challenges from the wildfires, measures that they will take for future wildfires, and measures that clinics and pharmacies should put in place. The clinician interview guide inquired about changes in their patients’ healthcare engagement after the wildfires; physical and mental health challenges of their patients as a result of the wildfires; other challenges regarding transportation, food security, housing, financial situation, and social isolation; programs that helped mitigate some of the effects of the wildfires; and any programs that can help support patients in the event of future wildfires.

One co-author (PS) conducted all interviews. Interviews lasted 60 min and took place on a HIPAA-compliant videoconferencing platform to allow for flexibility in scheduling and audio-recording of the interviews. All participants were offered a $40USD electronic gift card that was emailed to them.

Qualitative interviews were audio-recorded, transcribed verbatim, and field notes were created from each interview. We followed the procedures of Framework Analysis [38], a type of thematic analysis for qualitative data. We used both a deductive approach (based on patient and clinician interview guides) and inductive approach (based on emerging themes from the interviews) to develop our overarching themes for the matrix analysis. One investigator (KM) organized the research data into a framework matrix using Microsoft Excel, and two investigators (KM and PS) coded and identified overarching themes and findings. The investigators discussed data saturation, emerging patterns and themes, synthesized results based on patient and clinician responses, and selected exemplary quotes to further elucidate important discussion points.

## Results

Patients (N = 15) had a mean age of 56.7 years (SD = 7.8) and were majority male (93.3%), White (66.7%), and gay (60.0%) (Table 1). Most had some college education (40.0%), noted that they could barely get by on the money they had (66.7%), owned their place of residence (86.7%), and lived in a rural area of Northern California (73.3%). Mean time since HIV diagnosis was 23.7 years (range = 1–39 years) and 13 (86.7%) had an undetectable HIV viral load. Most reported fire or smoke damage to their place of residence (66.7%), disruption of transportation to and from medical services (53.5%), and disruption in food services (46.7%). Clinicians (N = 7) had a mean age of 51.0 years (SD = 14.2) and were mostly white (71.4%), cis-gender women (57.1%) (Table 2). Their professions included physician, nurse practitioner, registered nurse, pharmacist, social worker, and case manager. They had a mean of 21.4 years (SD = 18.2) of provision of direct patient care with a median of 155 patients with HIV on their panels.

**Table 1:** Characteristics of PWH participants

Characteristic	Responses	N = 15
Age, mean (SD)		56.7 (7.8)
Gender identity*, N (%)		
	Cis-gender man	14 (93.3)
	Cis-gender woman	1 (6.7)
	Genderqueer	1 (6.7)
Race, N (%)		
	African American	3 (20.0)
	American Indian or Alaska Native	1 (6.7)
	Multiracial/Multicultural	1 (6.7)
	White	10 (66.7)
Ethnicity, N (%)		
	Hispanic or Latinx	4 (26.7)
	Not Hispanic or Latinx	11 (73.3)
Sexual orientation, N (%)		
	Gay	9 (60.0)
	Bisexual	3 (20.0)
	Heterosexual	1 (6.7)
	Asexual	1 (6.7)
	Missing	1 (6.7)
Work situation, N (%)		
	Disabled, permanently, or temporarily	10 (66.7)
	Retired	2 (13.3)
	Working now (part-time or full-time)	2 (13.3)
	Looking for work, unemployed	1 (6.7)
Education, N (%)		
	High school or less	4 (26.7)
	Some college, no degree	6 (40.0)
	Associate or bachelor’s degree	4 (26.7)
	Master’s degree or higher	1 (6.7)
Financial situation, N (%)		
	I have enough money to live comfortably	3 (20.0)
	I can barely get by on the money I have	10 (66.7)
	I cannot get by on the money I have	2 (13.3)
Housing, N (%)		
	Own house/apartment/room	13 (86.7)
	Someone else’s house/apartment/room	2 (13.3)
Residential location, N (%)		
	Rural	11 (73.3)
	Urban	4 (26.7)
Time since HIV diagnosis, mean years (SD, range)		23.7 (11.2, 1–39 years)
Recent viral load count, N (%)		
	Undetectable	13 (86.7)
	Detectable	2 (13.3)
Impact from wildfires*, N (%)		
	Fire/smoke damage to residence	10 (66.7)
	Disruption in transportation to/from medical care	8 (53.3)
	Disruption in food services	7 (46.7)
	Disruption in other primary care needs	5 (33.3)
	Fire/smoke damage to a place receiving healthcare services (e.g., clinic/hospital/pharmacy)	5 (33.3)

* Total is greater than 100% due to ability to choose all options that applied

PWH: people with HIV; SD: standard deviation

**Table 2:** Characteristics of clinician participants

Characteristic	Responses	N = 7
Age, mean (SD)		51.0 (14.2)
Gender identity, N (%)		
	Cis-gender woman	4 (57.1)
	Cis-gender man	2 (28.6)
	Gender Non-binary	1 (14.3)
Race, N (%)		
	Asian	1 (14.3)
	Multiracial/Multicultural	1 (14.3)
	White	5 (71.4)
Ethnicity, N (%)		
	Hispanic or Latinx	1 (14.3)
	Not Hispanic or Latinx	5 (71.4)
	Prefer not to answer	1 (14.3)
Residential location, N (%)		
	Rural	3 (42.9)
	Urban	4 (57.1)
Primary profession/role, N (%)		
	Physician	2 (28.6)
	Nurse Practitioner	1 (14.3)
	Pharmacist	1 (14.3)
	Registered Nurse	1 (14.3)
	Social Worker (LCSW)	1 (14.3)
	Case Manager	1 (14.3)
Primary specialty, N (%)		
	Family Medicine	1 (14.3)
	Infectious Diseases	3 (42.9)
	Other	3 (42.9)
Years providing direct patient care, mean years (SD)		21.4 (18.2)
Total patients currently in panel per clinician*, median (IQR)		155 (50.0–256.3)
PWH currently in panel per clinician*, median (IQR)		155 (50.0–200.0)
Patients receiving HIV treatment per clinician*, median (IQR)		147.5 (47.3–196.8)
PWH impacted by wildfires per clinician, median (IQR)		25 (6.3–85.0)
Clinic impacted by wildfires in last 2 years, N (%)		
	Yes	5 (71.4)
	No	2 (28.6)

* Missing data from 1 facility

IQR: interquartile range; LCSW: Licensed Clinical Social Worker; PWH: people with HIV; SD: standard deviation

Five (71.4%) clinicians reported that their clinics had been directly impacted by wildfires in the past two years and they each had a median of 25 patients with HIV who had been impacted by wildfires (see Methods for definition of “wildfire impact”). All participants had been impacted by wildfires from 2015 to 2021 in various Northern California counties. Evacuations typically lasted from several days to many months.

### Living with HIV compounded by experiencing wildfires

Some patients felt that after living through the AIDS epidemic and losing many loved ones, they felt blessed to be alive and that these additional challenges of wildfires were just “one more little thing” in life. However, others worried about having a weakened immune system, remaining healthy under high stress, and having enough medication supplies during wildfires. Clinicians noted that patients experienced added vulnerabilities related to the compounding stress and trauma of wildfires after having already experienced multiple losses due to HIV over their lifetime.

“It feels like there’s been community trauma after community trauma and it all kind of runs together. It almost feels like for the last couple of years, you just can’t get a break, patients feel that. We’ve had some upswings where things felt a little better and then another fire hits or another evacuation hits or another power outage.” (P03, clinician)

While some clinicians noted no change in virologic suppression among clinic patients, others stated that they suspected that patients who were unsuppressed were also the individuals who had fallen out of care and had not had a viral load checked. One clinician stated that there was a considerable reduction in virologic suppression in their clinic after the wildfires. Patients also noted that they had experienced an increase in their viral load due to stress or inability to maintain a routine that included daily treatment adherence while evacuated.

**Fig. 1:**
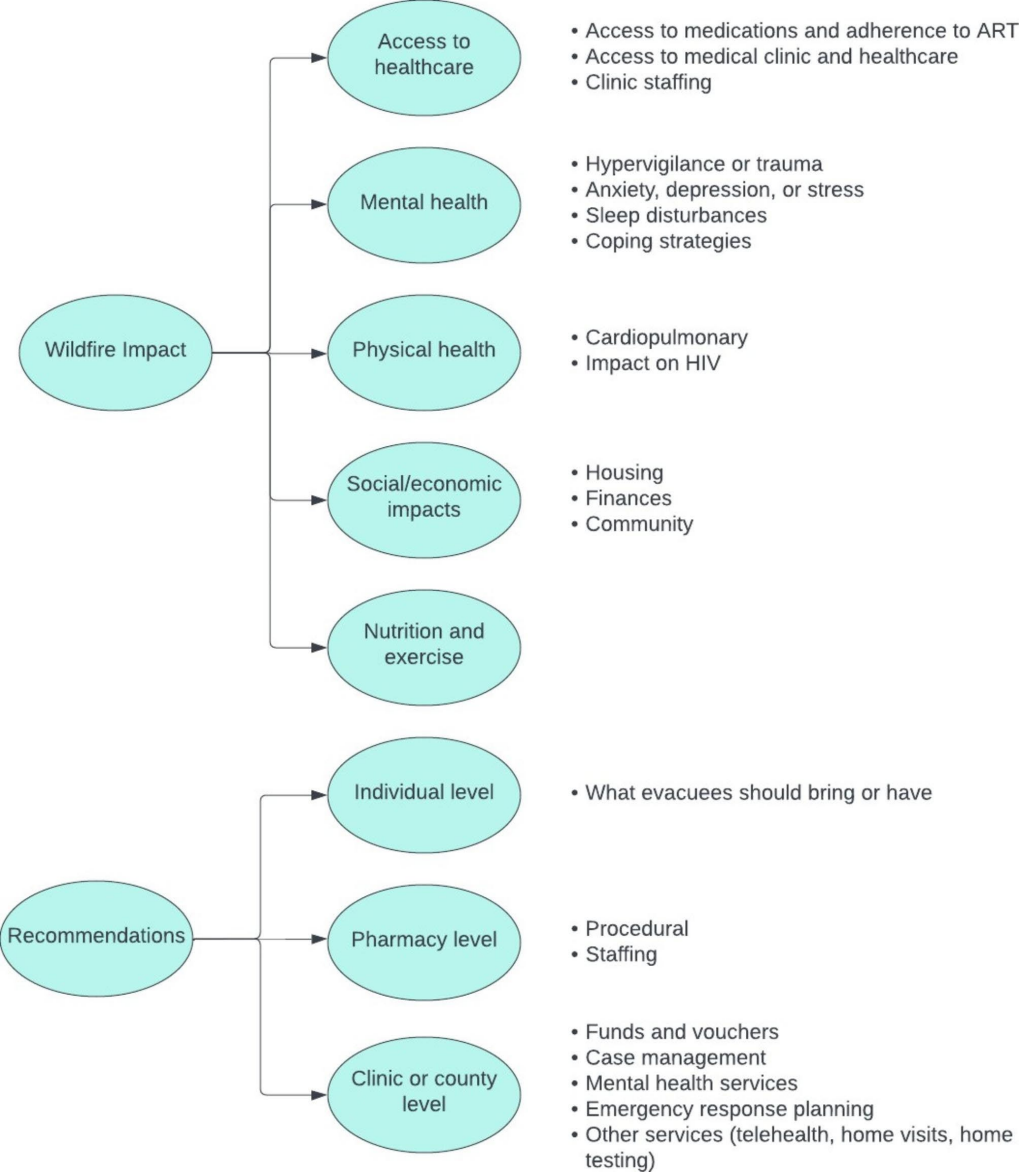
Themes and sub-themes of qualitative interviews with patients with HIV impacted by wildfires and their clinicians

Below we detail findings related to wildfire impact and recommendations for future wildfires. These findings are summarized in Fig. 1.

### Wildfire impact

Participants outlined five main routes by which wildfires negatively impacted their health: **(1)** access to healthcare (medications and adherence to ART, medical clinic and healthcare, and clinic staffing), **(2)** mental health (hypervigilance or trauma; anxiety, depression, or stress; sleep disturbances; and coping strategies), **(3)** physical health (cardiopulmonary and other co-morbidities), **(4)** social/economic impacts (housing, finances, and community), and **(5)** nutrition and exercise (Fig. 1).

#### (1) Access to healthcare

This topic included sub-themes related to access to medications and adherence to ART, access to medical clinic and healthcare, and clinic staffing.

#### Access to medications and adherence to ART

Participants generally reported few pharmacy or ART adherence barriers prior to wildfires. During and after the wildfires, many reported difficulties with ART adherence and maintaining access to medications, resulting in needing to stockpile medications. The main reasons for missed doses included depression and stress related to wildfires, having a change in routines, and evacuating in a rush and forgetting medications. Patients also noted not wanting to leave evacuation areas to pick up medications for fear of not being able to return home. One patient noted that he missed six days of medications because he would have to drive 4.5 h to get to the pharmacy due to road closures and that he worried that he would not be allowed to come back home due to mandatory evacuations. A clinician recalled patients having to hide medications from family members with whom they were staying during the wildfires.

Some system level barriers during wildfires included the lack of functioning of medication mail delivery, closure of many pharmacies, pharmacies running out of medications, inability to convince insurance to authorize extra refills, and insurance not covering medications in another state to which the individual had evacuated. Conversely, some system-level factors facilitated medication access. These included the ability of clinicians to authorize refill requests via clinic mobile health apps or electronic health record systems even during clinic closures, assistance of clinic staff to link to nearby pharmacies, use of pharmacy emergency waivers to refill medications for chronic conditions without a provider refill authorization, a mobile pharmacy at evacuation sites, clinic staff being permitted to enter the evacuation zone to deliver medications, and expedited medication delivery. During the wildfires, patients reported deeply appreciating that some pharmacies understood they had lost everything, took care of authorizing early refills, and drove medications out to them within two days of evacuation.

#### Access to medical clinic and healthcare

During and after the wildfires, some clinics were closed for weeks due to smoke or power outages, including a rural HIV clinic that only occurred quarterly, and one clinic was lost completely due to smoke and water damage. During this time, some clinicians were able to redirect their patients to satellite clinics to receive care, and the clinic that was lost was later able to see patients for urgent visits by setting up trailers in the parking lot for exam rooms.

Clinicians noted difficulty contacting patients as landlines were disconnected, emergency contacts had also been evacuated, and many patients did not use email for communication. Some clinic staff reached out to patients by calling them, sometimes daily, after wildfires to provide social support. Clinicians also noted decreased engagement in HIV care during wildfires, and that clinical care had evolved to acute care with increases in emergency room visits.

Transportation during and after wildfires became challenging as road closures and construction extended travel times from 30 min up to 2 or more hours. Clinic shuttles that had assisted patients prior to wildfires stopped operating during wildfires and drivers were sometimes tasked with helping people evacuate. Clinicians and patients both recalled transportation barriers related to the air being too smoky to take public transit, inability of patients to afford ride-hailing services due to loss of work, and patients no longer being able to rely on friends for help with rides to clinics due to being evacuated themselves or not wanting to be outside in the smoke. Patients did not find enough value in receipt of clinical care to withstand the long smoky commute.

“And then the fire happened and the roads started to close, and that was the biggest barrier of people being able to access medications, or going to doctor’s appointments or anything… So usually, a trip that would be a half hour… would be 4.5 hours because they would have to do a complete loop around.” (P02, clinician)

Telehealth options varied, with some clinics conducting mostly telephone visits given that clinicians did not have a private space to use video during the visits. Some patients reported difficulty with telehealth due to not having a private space to talk at home especially if staying with family during evacuations or not having access to Wi-Fi.

“One patient had to move his [evacuated] mother into the house with him and it made what was a comfortable house [seem] very very small. And she didn’t know his [HIV] status, so he couldn’t do telephone appointments or have his medications where they normally were and things like that.” (P04, clinician)

#### Clinic staffing

Prior to wildfires, clinics had the challenge of maintaining staff in a rural area. Patients reported that there were limited appointments due to a shortage of infectious disease or HIV specialists in the area. This challenge was further exacerbated during and after the wildfires due to employees being evacuated or moving away from these areas. This shortage translated to clinics becoming busier and less responsive to patient needs due to being overburdened.

#### (2) Mental health

Patients reported increased hypervigilance or trauma; anxiety, depression, or stress; sleep disturbances; and coping strategies that they used to deal with mental health challenges.

#### Hypervigilance or trauma

Patients reported being hypervigilant and being triggered by the sounds of text emergency alerts, fire engines, and the smell of smoke. They noted a feeling of always looking over their shoulder, acting like “a cat on a hot tin roof,” and tracking nearby fires. Patients recounted the sentiment of being uprooted, a lack of control after being displaced with nothing, and not wanting to buy anything after losing everything. Clinicians described the wildfires as compounded trauma from multiple fires and noted that the cyclical nature of the wildfire season has been especially traumatizing for people who were unhoused, people who had fled war-torn countries, and people who had experienced multiple losses in their lifetime. One clinician noted her patient’s description as a “triple whammy” (i.e., HIV, wildfires, and COVID-19). One patient recalled the wildfires as being more traumatic than the 1989 Loma Prieta earthquake and the toll of the AIDS epidemic.

“I was in the earthquake in ‘89 in San Francisco, people died in my office. I went through HIV in San Francisco. This was different. It was surreal but it was traumatizing… you can’t believe it’s happening. And I remember when we came back [from being evacuated due to a wildfire]… and when we turned the corner, all the houses on our courtyard, they were all gone, everything was gone. It changed everybody’s life.” (W10, patient)

Clinicians noted that while patients felt frustrated by the COVID-19 pandemic, many drew on their experience with HIV and had an understanding of what a virus is, how to assess risks versus benefits, how to cope with stigma and shame, and felt some control over what they could do to keep themselves healthy. Conversely, they felt a lack of control with the wildfires and that the wildfires took away their access to nature, which was a reason many patients said they had moved to these less urbanized areas.

#### Anxiety, depression, or stress

Patients reported feeling anxious, depressed, and stressed. These mental health challenges were multi-faceted and often health-related, social, financial, or environmental. Health-related worries included the potential for lung damage from wildfire smoke, losing or forgetting to bring valuables when evacuating, or not having enough medications.

“When they said it’s a bad air day, I didn’t want to take the risk so I just wouldn’t go out. I had never been that depressed. I had never been in a situation where I couldn’t go outside freely when I wanted to. It just caused me a lot of feeling down and out, a hopeless feeling. What is the purpose of life if I can’t breathe air? Why am I here? It was like I was being tormented, like something was haunting me.” (W03, patient)

Some mental health challenges were associated with social issues such as staying with other people during evacuations, experiencing a lack of privacy, or feeling isolated. Some mental health challenges were environmental, such as not knowing whether it was safe to go outside and the anticipation of when the next fire season would start. Other challenges were financial and related to evacuation from or destruction of their home, such as difficulty in finding affordable housing, the threat of homelessness, or losing independence.

“[Being evacuated] was the most stressful situation I’ve ever been in. It drove my anxiety through the roof not knowing if I was gonna have a home to come home to or not. All I had was what I had in the car. Thinking like wow, ‘We’re gonna really end up homeless out here with me, my mom, my brother, and a dog?’” (W14, patient)

Patients noted feelings of constant uncertainty, hopelessness, lingering anxiety, and felt that “nowhere is home” after being displaced from their original communities. Clinicians noted an increase in requests for anti-anxiety medications and reported that patients had a sense of unfairness after having already lost or dealt with so much.

“…it’s so complicated, I think a lot of long term survivors assumed they weren’t going to be around that long, and when coming out of a time that was so dark, where so many people died, loved ones, lovers, family members, sometimes I feel like I see a detachment from the practical, like wanting to celebrate, understandably, life and decadence… ‘this cigarette is the last vice I have, let me have it’, things like that. There’s a hesitation to ask people to do more than they’ve already done, I think.” (P03, clinician)

#### Sleep disturbances

Patients reported interrupted sleep, insomnia, nightmares, experiencing a racing mind when trying to sleep, not having a stable place to sleep, not sleeping well in new places, or sleeping more due to depression. They also noted increased marijuana use for sleep.

“…it’s a big change from before. I used to sleep all the time, at the drop of a hat I could fall asleep. But since these wildfires, my brain is just racing. I would try to close my eyes, my brain is constantly going and going. It’s not fun. I’m walking around all night, sitting around watching television, or just sitting outside. For hours.” (W02, patient)

#### Coping strategies

Some clinicians noticed increased use of substances such as methamphetamine, opiates, and alcohol among their patients, as a way to cope. Patients reported challenges with their usual coping strategies due to inability to exercise or socialize due to the pandemic and the smoke and not having access to nature.

“One big source of support for people here is nature, a lot of people moved up here for the mountains and whatnot. Wildfires took that away, and now you fear it. That adds insult to injury, and then you have to live with the burn scars where fires were. That damage lasts for a lifetime, you can’t get away from it, you see those reminders.” (P06, clinician)

Patients noted using other coping strategies such as breathing exercises, distracting themselves with work, online support groups, behavioral health visits, talking to case managers, and anti-anxiety medications.

#### (3) Physical health

Patients and clinicians discussed physical health vulnerabilities related to cardiopulmonary sequelae, other co-morbidities, and urgent care use.

During and after the wildfires, many patients reported difficulty breathing, increased coughing, wheezing, exacerbated asthma, lung involvement, taking prednisone, starting or increasing inhaler use, and increased blood pressure. Similarly, clinicians noted that patients with prior stable or distant diagnoses of asthma and chronic obstructive pulmonary disease had exacerbations in their condition. They noted that the smoke from the wildfires had the most significant impact on patients’ cardiopulmonary health given that most patients were older and had multiple comorbidities. Clinicians reported that patients who had prescriptions for inhalers were refilling more often, and there was approximately a quarter percent increase in inhaler requests. As a result, clinicians noted that urgent care visits were mostly related to respiratory complaints and inhaler requests. Overall, cardiopulmonary and other co-morbidities were noted as added physical health vulnerabilities that patients had to deal with in addition to HIV care.

**(4) Social/economic impacts**: Participants discussed the impacts of wildfires on their housing, finances, and community.

#### Housing

Patients discussed the impacts of wildfires on their homes, including having smoke damage or losing everything they owned. Some patients were able to rebuild their home or moved to a new house nearby, while others moved to another region or were simply lost to clinic follow-up. Clinicians noted that housing resources had already been scarce and were worsened after wildfires. They stated that long-term housing for displaced patients was hard to come by, since many individuals displaced from previous surrounding fires were also looking for housing. As a result, people were priced out of the local housing market following the wildfires and there was an increase in unhoused or unstably housed patients. Additionally, clinicians noted that some patients were without water, power, or gas for weeks.

#### Finances

Patients reported significant financial burdens due to having to purchase food and supplies during evacuations, supporting friends and family who had been evacuated, paying for out of state hospital bills that were not covered by insurance, and paying for short-term hotels or rentals. Many reported price gouging from hotels and rentals and, for those who had lost their homes, having to contend with fraudulent contractors. They discussed struggling with loss of work or business, workplace closures, and finding work during the COVID-19 pandemic.

“I couldn’t get a job because I didn’t have a computer. I didn’t have a good phone. I didn’t have money, so I got a government phone, and I realized that I was a person in society that did not have access to technology. I couldn’t go to the public library because it wasn’t open. Later on in the pandemic, it was open, but you couldn’t use the computers because multiple people would be touching them. And that became so frustrating. To realize that a person whose livelihood is based on writing can’t write.” (W04, patient)

Clinicians noted that some patients changed jobs due to job loss or employers closing down due to fires, and others had to take on more shifts as they struggled financially to try to make ends meet. While some patients had support from renter’s insurance, others took on credit card debt to get through.

#### Community

Patients recalled that their communities were devastated. They were not able to gather outside due to smoke and that many neighbors moved or were displaced to different areas. Patients were also disconnected from friends due to road closures. Patients noted that they often heard about approaching wildfires, looming evacuation orders, or lifting of evacuation orders from neighbors. These notices often occurred well before official notifications or orders from safety officers.

#### (5) Nutrition and exercise

While one patient noted weight loss due to stress, others reported weight gain during and after the wildfires due to inability to go outside to exercise, increased snacking, and eating mostly fast food while evacuated. Those who did not evacuate during wildfires, reported eating mainly canned or dried food (recounted as being on a “pop tart diet” by one patient) because they did not want to leave the evacuation zone for fear of inability to return to their home.

“It was the worst to have to depend on Doordash and fast food [while being evacuated]. It was hard to find decent stuff to eat that wasn’t fattening or high in salt or sugar…. Even after we were able to come back in town, it was a week or two before the stores and gas stations were able to open up because we couldn’t get trucks up here. We had to depend on what we had left in our freezers and refrigerators. We had to throw out most of the meats and milk and dairy products [because they were expired]....” (W14, patient)

They also noted that stores had run out of food due to road closures, that food banks were closed for several consecutive weeks, and that they lost refrigerated foods due to power outages. Unemployment during wildfires and the COVID-19 pandemic further exacerbated food insecurity.

### Recommendations for future wildfires

The recommendations for future wildfires are organized at multiple levels (Fig. 1): on the **(1)** individual level (what to bring or have), **(2)** pharmacy level (procedural, staffing), and **(3)** clinic or county level (funds and vouchers, case management, mental health services, emergency response planning, and other services [telehealth, home visits, home testing]).

#### (1) Individual level preparedness

These recommendations included tips on what evacuees should bring or have.

#### What evacuees should bring or have

In addition to non-medically-related items, such as passport and identification cards, cash, clothing, pet supplies, food and water, and telephone and charger, patients noted the importance of having their prescription and over-the-counter medications, health insurance information (including AIDS Drug Assistance Program [ADAP] paperwork), and other health-related paperwork (such as advanced directive) during evacuations. Especially during fire season, most had a ready-packed bag with many of these items in case of an emergency evacuation. Clinicians also stated that it was important to take a medication list with copies of prescriptions (e.g., photos of prescription bottles) in case of the need for refills at another pharmacy. Patients noted that they built up a stock of medications by refilling early or asking for overrides so that they could have several weeks of medications in case of an evacuation or inability to reach the pharmacy.

#### (2) Pharmacy level preparedness

These findings related to procedural issues and staffing preparedness.

#### Procedural

Patients and clinicians noted it would be helpful if the health insurance companies could allow for three-month supplies of medications (especially ART) and authorize early refills to ensure patients always have a stock of medications during wildfires. They also suggested that pharmacies should help patients set up medication delivery, provide one-week supplies of medications in case of emergencies, and have a protocol on how patients can access medications in emergency situations. One clinician recounted the benefit of a mobile pharmacy that was set up at an evacuation center to help patients access their medications. Clinicians also noted that state-specific emergency waivers [39] may be used by pharmacists during disasters to fill necessary medications without the approval of a physician.

“I must have been a little liberal with some of the directives, but when somebody would show me their bottle and it was from another pharmacy, I would just create a prescription from that and fill it for them. You just have to document that you’re using the waiver to fulfill x, y, z. So now because of all these fires and floods and pandemics and waivers, we have an actual emergency board of pharmacy binder where we document when we’ve gone off your traditional law and used the waiver to do certain things.” (P08, clinician)

#### Staffing

Clinicians noted that having support from the pharmacy to hire skilled technicians could make a difference in patients’ medication access. One clinician noted the importance of hiring and training pharmacy technicians who can proactively communicate with clinicians about patients’ medications and who are knowledgeable about mechanisms for health insurance overrides and emergency refills.

“I don’t think it’s anything special about [pharmacy] except that our technicians are amazing and they know how to call the insurance and get an override for whatever reason, if it’s evacuation, lost medication, vacation supply… It’s so sad to me, how basic care like this deteriorates [during emergency situations such as wildfires]. I mean, is it really going to break [the chain pharmacy’s] bottom line [to hire 1 to 2 more technicians]?” (P08, clinician)

#### (3) Clinic or county level preparedness

Participants discussed clinic or county level preparedness including funds and vouchers, case management, mental health services, emergency response planning, and other services such as use of telehealth, home visits, and home laboratory testing.

#### Funds and vouchers

Many patients used emergency vouchers and donations to help pay for clothing, food, gas, bills, hotels, and pet supplies. While these needs were met, patients also noted it would have helped to have funds for air purifiers, internet access, telephone, and computer. Clinicians also noted that having had access to unrestricted funds to assist patients with unique financial needs and patients who were evacuated across state lines would have greatly improved care especially in rural areas.

“People making these [restricted funding] decisions are probably sitting in an office in some big city and they don’t know, it doesn’t even cross their mind [to provide patients with funds to assist with specific financial needs or out of state expenses]. I wish they would talk to someone from a rural area [that has been impacted by wildfires].” (P07, clinician)

#### Case management

Clinicians noted a need for expanded case management to help patients with “bureaucracy literacy.“ Patients reported that the most helpful programs, such as Ryan White Programs, offered wraparound services where a social worker or case manager spoke with them to fully understand their needs and help them through the process of accessing resources such as food, medical care, housing, telephone, vouchers, and mental health services. This included the clinic staff who proactively outreached to all their patients to inquire about their health and their whereabouts, and to alert them to locations to receive services and clinical care in the wake of wildfires. Patients contrasted this to programs that provided flyers for services. These programs were referred to as “job fairs” that provide lots of flyers but were less helpful in understanding the needs of an individual and tailoring the response.

“They set up all these tables with all these agencies, like how to get fire insurance. You’d leave with this tote bag with all this paper, with all these websites, all these phone numbers that you didn’t use because you didn’t have a phone or a computer, or you’re so frustrated, you’re so sad, you’re so in need, you just throw it all away.” (W04, patient)

#### Mental health services

Clinicians noted that the mental health impacts of the wildfires were the main challenge that they dealt with and described patients as now having “an underlying baseline feeling of heightened awareness and anxiety.” They stated that it was helpful to have consistent access to mental health programming and professionals, such as on-call mental health staff, emergency counseling services, therapists who could visit shelters, and online or in-person support groups.

#### Emergency response planning

Patients requested education on fire preparedness, guidance on what to stock in their go bags, earlier warning of when an evacuation may occur, and information on emergency resources and programs. County alerts and websites, clinic updates posted to social media, calls from clinic staff, and communications with neighbors were some of the mechanisms by which patients received information. Backup generators, text-message alerts, phones trees, and an operations team that supported clinic employees helped clinics communicate with staff during wildfires. However, clinicians noted the need for clinics to plan ahead for natural disasters and better mechanisms to communicate with patients.

#### Other services

Telehealth visits with clinicians, flexible hours for in-person visits, home visits, emergency transportation options, home laboratory sample collection, connecting patients to satellite clinics or nearby pharmacies and laboratories, and use of long-acting injectables were examples of other services that clinics could implement now to improve response during future wildfires. Some patients also noted the need for support to access and use mobile health technology, for example classes for using the clinic mobile app or video-counseling software.

## Discussion

Few studies have looked at how wildfires uniquely impact PWH. In this qualitative study, we noted that, while some patients felt that surviving the HIV epidemic added to their resilience against wildfires, many felt that the wildfires compounded their trauma and anxiety. This was particularly true for those who had unstable housing, lacked family or community support, or lacked financial means. Patients worried about having enough medication supply, maintaining adherence during an evacuation, living with underlying health vulnerabilities, and stigma and privacy concerns. Many patients pointed out the unique struggles of living in a rural area. These included lack of access to nearby HIV specialists, limited Wi-Fi access, and lack of public transit and transportation. During and after the wildfires, these challenges were exacerbated due to smoke, power outages, and road closures, which also limited the ability to receive food and emergency supplies. Furthermore, emergency funding was restricted and did not take into account unique needs of evacuees and mobile populations, such as access to healthcare and medications across state lines. Finally, participants noted mental health impacts as being one of the biggest and longest lasting sequelae of wildfires.

Given the impact of wildfires on health and healthcare access, there is a clear need for mitigating their destructive effects and instituting adaptive interventions to shore up healthcare infrastructure. Participants suggested increasing the availability of clinic mobile health apps to allow clinicians to authorize refill requests remotely during clinic closures, creation of informational material about nearby clinical services and pharmacies, increasing pharmacy emergency waivers allowing for medication refills without prescriber authorization, use of mobile pharmacies at evacuation sites, and expedited medication delivery. Additionally, implementing policies to extend health insurance coverage across state lines during EWEs is important. Other key strategies include increasing pharmacy staffing in rural areas, as well as training clinic staff and pharmacy technicians on emergency protocols to provide timely services, medication, and information to patients during EWEs. Lastly, making it easier for rural populations to access infectious diseases and HIV specialists through supporting telehealth options, or through providing the tele-consultations for rural-based clinicians to consult with HIV specialists based elsewhere (e.g., the National Clinician’s Consultation Center’s HIV Warmline) can help address inequities among vulnerable populations and in rural areas.

In 2020, wildfires in the West Coast of the US burned over 2.7 million hectares, killed more than 30 people, left tens of thousands of people without a home, and resulted in over $20bn one-year economic impact [40]. According to the First Street Foundation [41], by 2052, 56% of all addresses in the lower 48 states will face some degree of wildfire risk. They estimate that over the next 30 years in the US, nearly 20 million properties face “Moderate” risk, 6 million properties face “Major” risk, 3 million face “Severe” risk, and approximately 1.5 million face “Extreme” risk of experiencing a wildfire [42]. In California, the site of the present study, this impact has already been felt, with seasonal wildfires intensifying in the past decade. Twelve of the 20 largest wildfires in the state’s history have taken place in the past 5 years, and the state continues to experience heatwaves [43, 44]. These climate-driven changes also have structural and health impacts that have farther reaching effects. The challenges associated with wildfires will continue to increase and require more attention and resources over the next few decades.

In addition to the direct impacts of wildfires, smoke from wildfires have been associated with negative mental health and wellbeing impacts, including increased anxiety, depression, isolation; lack of motivation; and respiratory issues [27]. A systematic review of wildfires and sleep disturbances suggested that sleep disturbances are highly prevalent in wildfire survivors, with insomnia prevalence as high as 63–73% and nightmares ranging between 33 and 47%. This review highlights the associations between sleep disturbance prevalence, post-traumatic symptoms following the trauma of wildfires, and the severity of and proximity to wildfires [45].

Several studies have demonstrated the link between wildfires and mental health including anxiety, PTSD and suicidal ideation [22, 46, 47]. A recent scoping review of the mental health impacts of wildfires found that wildfires were associated with increased rates of PTSD, depression, and generalized anxiety directly after wildfires and years after [22]. Poor mental health, in turn, has been shown to drive worse engagement in HIV care and treatment [48]. Substance use, such as alcohol and drug use are also associated with poor ART adherence [49, 50]. It is possible that PWH may turn to substances to cope with the negative mental health impacts of wildfires, as has been suggested by our data, with ultimately detrimental impacts to their ART adherence. These mental health impacts, as well as other economic and social impacts of wildfires, are inequitably distributed based on vulnerabilities related to income, occupations, age, preexisting health conditions, housing status, and social isolation. Ultimately, wildfires can exacerbate existing health disparities. Low-income individuals, those living in rural and/or resource-limited settings, and other vulnerable populations will likely experience the effects of EWEs with more intensity than high-income individuals living in urban and/or high-resource settings.

**Fig. 2:**
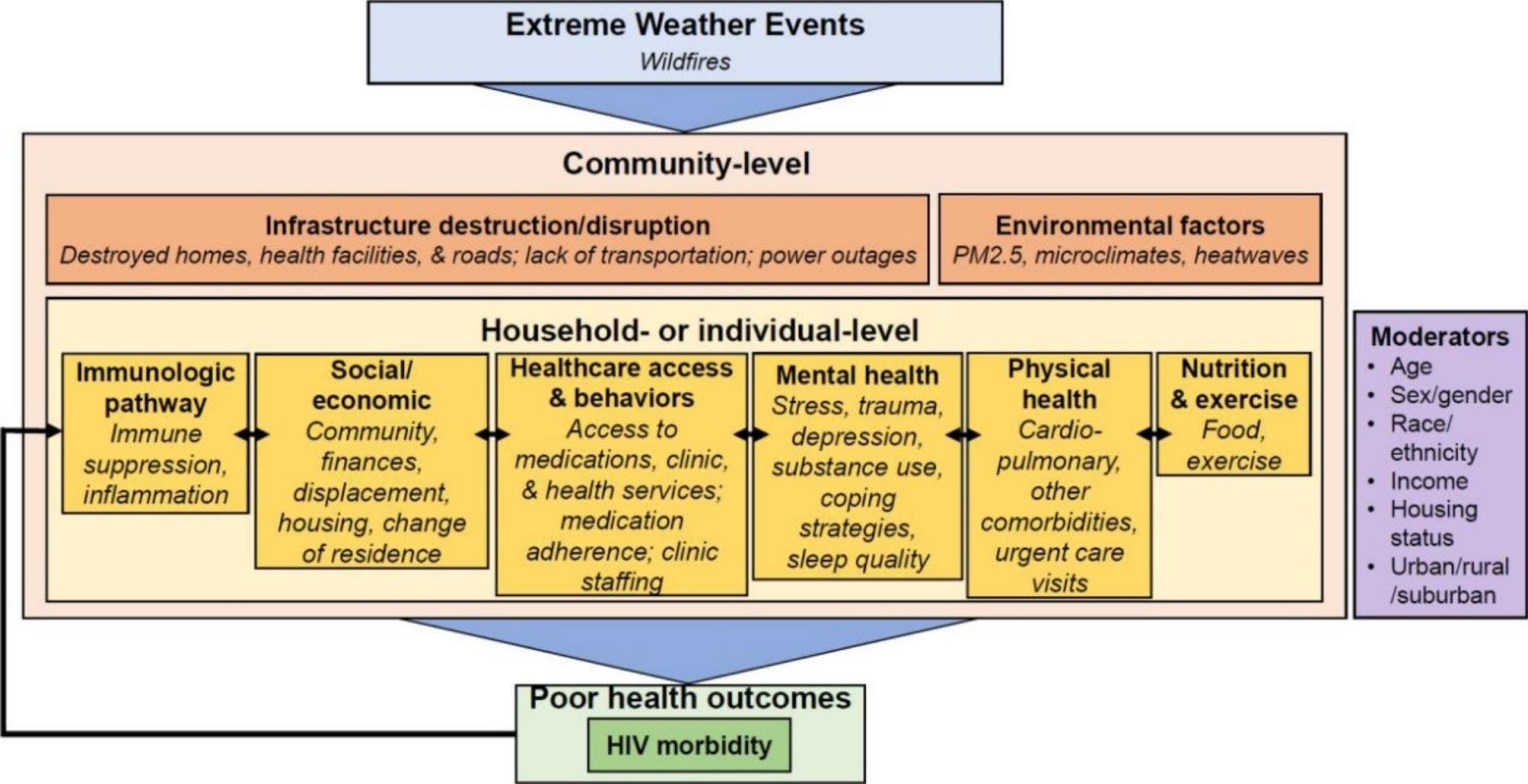
Conceptual framework of the pathways between wildfires and poor health outcomes among people with HIV

Based on our data and prior research, we have devised a conceptual framework to capture the pathways between wildfires and health outcomes among PWH (Fig. 2). The proposed conceptual framework also uses information from a conceptual framework of climate change and HIV in Sub-Saharan Africa [51] and a syndemic framework for HIV and the environment [52]. The proposed framework acknowledges that wildfires impact aspects of the community-, household-, and individual-level with implications for a variety of health outcomes. At the community-level, wildfires can disrupt or destroy infrastructure necessary to access basic resources and health care, damaging physical and communications infrastructure as well as impacting health care personnel themselves [53]. At the community-level, wildfires have been shown to impact and be impacted by other environmental factors. Other environmental factors impacted by wildfires include elevated levels of particulate matter 2.5 (PM_2.5_) in the air due to smoke and microclimates indicators (e.g., land surface temperature and normalized difference vegetation index) which are also associated with poor health outcomes [54, 55].

Although we were unable to directly assess the immunologic pathway through which wildfires exacerbate HIV-related health outcomes in our qualitative study, wildfires have been shown to lead to immune suppression and inflammation [56, 57], which are associated with HIV morbidity and mortality [58]. Therefore, this suggests one pathway that requires further investigation in future research. In addition to HIV-associated morbidities, these pathways can impact poor health outcomes related to cardiopulmonary and other comorbidities. Ultimately, our framework posits that the link between wildfires and poor health outcomes work in a vicious cycle, whereby individuals experiencing wildfire-induced/exacerbated HIV morbidity may experience even greater barriers to treatment adherence and poor mental health outcomes. The dynamics outlined in this framework are moderated by gender, age, income, race/ethnicity, housing status, and residential location (urban/rural/suburban).

Our research has several limitations. The duration of the wildfires overlapped with the COVID-19 pandemic, as a result, some of the reported challenges may have been exacerbated by the impact of the other. However, given that our study focused mainly on EWEs, we could not make any conclusions about how COVID-19 could fit into our conceptual framework. Additionally, participants were located in Northern California and therefore results may not be generalizable to other PWH in other areas.

## Conclusions

In summary, our qualitative study reveals important information about the intersection of two extreme global public health challenges that particularly affect vulnerable populations. These data help shed light on the pathways through which wildfires impact health outcomes among PWH and can help in developing interventions, programs, and policies to mitigate the cumulative impacts of EWEs on the health of PWH, particularly among those in rural areas. Understanding vulnerabilities, adoption of health system strengthening strategies, and improving community resilience through disaster preparedness is needed more than ever.

## Data Availability

The datasets generated and/or analyzed during the current study are not publicly available due to the fact that we interviewed a small number of clinicians in Northern California whose patients were impacted by the wildfires and who talked about their experiences with local facilitators and barriers; therefore, sharing of data may result in the disclosure of their identities. However, data may be available from the corresponding author on reasonable request.

## Abbreviations

ART: Antiretroviral therapy
COVID-19: Coronavirus Disease 2019
EWE: Extreme weather event
PWH: People with HIV
